# Reductions in anxiety, depression and insomnia in health care workers using a non-pharmaceutical intervention

**DOI:** 10.3389/fpsyt.2022.983165

**Published:** 2022-09-06

**Authors:** Katherine Currie, Babu V. Gupta, Ishan Shivanand, Amit Desai, Shweta Bhatt, Hari S. Tunuguntla, Sadhna Verma

**Affiliations:** ^1^University of Kentucky College of Medicine, Lexington, KY, United States; ^2^Neuropsych Center of Greater Cincinnati, Sharonville, OH, United States; ^3^SYC Infinite, San Francisco, CA, United States; ^4^Mayo Medical Center, Lucknow, India; ^5^Rutgers-Robert Wood Johnson Hospital, New Brunswick, NJ, United States; ^6^The Cincinnati Veterans Administration Hospital, University of Cincinnati College of Medicine, Cincinnati, OH, United States

**Keywords:** depression, healthcare workers, anxiety, insomnia, mental health, mindfulness, yoga

## Abstract

The COVID-19 pandemic has caused significant medical and psychological challenges worldwide, and not only exceeded the capacity of hospitals and intensive care units but also an individuals’ ability to cope with life. Health-care workers have continued to provide care for patients despite exhaustion, fear of transmission to themselves and their family, illness or death of friends and colleagues, and losing many patients. They have also faced additional stress and anxiety due to long shifts combined with unprecedented population restrictions, including personal isolation. In this study, we study the effect of an app-based Yoga of Immortals (YOI) intervention on mental health of healthcare workers. In this study, the health care workers were digitally recruited, and their psychological parameters were measured using validated questionaries. The participants were randomly grouped into control and test groups. The validated psychological measures were the Patient Health Questionnaire-8 (PHQ-8), Insomnia Severity Index (ISI) and generalized anxiety disorder (GAD-7) scales. The digital YOI intervention significantly reduced the anxiety, depression symptoms, and insomnia in healthcare workers of all age groups. In contrast, there was no improvement in the control group. This study details the effectiveness of an app-based YOI intervention in healthcare workers.

## Introduction

The COVID-19 pandemic exacted a profound emotion, physical, and economic toll on the medical system as well as the entire range of healthcare workers (1–3). Care delivery systems around the world have faced relentless demand, with front-line staff risking their own lives due to exposure. Even before the COVID-19 pandemic, there were multiple issues with the healthcare system, including pressure on providers from working long hours and facing inherent dangers all without proper resources, or clarity on the limits of their duty of care (4). Unsurprisingly, the pandemic has exacerbated these issues, leaving health workers facing burnout and fear of infection, as well as isolation from their families and other support structures (5).

Recent literature has quantified the extent of the emotional and psychological toll on healthcare workers. A review and meta-analysis by Pappa et al. included over thirty thousand healthcare workers, and found that at least 23% of workers exhibited anxiety, ∼22% met criteria for depression, and over 38% experienced insomnia (6). Another study of mental health workers in China found rates of depression, anxiety, and insomnia of ∼50, ∼45, and ∼34%, respectively (7). Furthermore, Sinsky et al. conducted a survey, which found that 2 in 5 nurses and 1 in 5 doctors reported their interest in leaving their profession as a result of the COVID-19 pandemic (8).

These negative effects, are reported in the literature, can impact not only mental health but care quality as well. Healthcare workers operating with psychological stress and poor mental health tend to have an increased incidence of medical errors (9). These errors are estimated to cause over 90,000 deaths and cost over $19 billion per year in the United States (10). Given the tremendous cost of such errors, and the role that healthcare practitioner stress plays in driving them, there is a compelling societal interest in developing interventions targeting the emotional needs of healthcare workers who are facing a public health crisis.

Before such interventions can be implemented on a wide scale, careful research and clinical trials are needed to test the efficacy of any intervention seeking to improve emotional well-being and mental health. While studies on psychological interventions for healthcare practitioners during such crises exist (11–17), many of these studies (a) are underpowered, (b) lack proper control groups, or, potentially most importantly, (c) consist of interventions that may be effective but are not scalable and easy to rapidly implement during a crisis. For example, cognitive behavioral therapy (CBT) interventions for healthcare workers have been studied in the context of the Ebola epidemic (18). A group of UK-based clinicians traveled to Sierra Leone to train hospital staff on administering CBT to their colleagues. Significant improvements in mental health indicators, including questionnaires on anxiety, insomnia, and substance use, were observed over the course of the intervention. While suggestive, this study lacked a control group. Further, the nature of the intervention, in which clinicians travel to train local hospital staff in CBT, is simply not feasible during a global pandemic, in which travel is limited and hospital capacity is sufficiently stretched that there are no staff to spare for CBT training. There remains a need to explore novel and innovative treatment modalities that are easily available and are cost-effective, and which ideally do not require in-person contact.

One such novel treatment, which can be implanted in a cost-effective manner that does not rely on in-person meetings, in Yoga of Immortals (YOI). The YOI intervention is an educational program that teaches specific practices based on ancient yogic teachings (19, 20). The core components of the YOI program—breathwork, whole body movements, and postures—have been shown to have multiple emotional benefits. The literature has shown that breathwork, a core concept of the YOI, can influence the autonomic nervous system function, including vagal tone, through the voluntary control of breathing patterns (21). Specifically, a recent clinical trial (22) found that four weeks of Sudarshan Kriya yoga, a practice focused on breathwork, reduced measures of psychopathology in patients with generalized anxiety disorder. Another recent randomized clinical trial (23) also reported significant improvements in both psychological and physical symptoms after six weeks of a “Breath-Body-Mind Workshop,” which teaches some breathwork along with whole body movements and meditation. Psychophysiologically, the reduction in depressive symptoms observed following breathwork, such as abdominal breathing, appears to be associated with a reduction in cortisol levels (24). Abdominal breathing may serve as a bridge linking the autonomic nervous system and the central nervous system to mobilize vagal activation of GABA (gamma-aminobutyric acid) pathways from the prefrontal cortex and insula, and to inhibit amygdala overactivity (25), leading to decreased depressive and anxiety symptoms.

Though yoga and meditative practices are associated with numerous physical and mental health benefits (26), one potential drawback is that such practices are mostly taught through scheduled in-person sessions. This limits their availability, during a pandemic, when group gatherings are inadvisable. A structured YOI program was developed for a mobile platform in the hopes of reaching a wider swath of the population. Previous work by our group has demonstrated that this YOI program has a beneficial effect on psychopathology in broad samples of the population, even during periods of elevated stress such as the COVID-19 pandemic (19, 20, 27). The YOI program has many of the desired characteristics of an ideal mental health intervention for stress in healthcare workers, including (a) a strong evidence base; (b) scalability, in that it is distributed via a low-cost app and does not require a trained clinician; (c) flexibility, in that healthcare workers with onerous workloads can pursue the program as their schedule allows; and (d) compatibility with any social distance or quarantine policies that may be in place, given that the program is completed alone.

To test the efficacy of an intervention involving yoga and meditative practices, the present randomized controlled study investigated the effect of the YOI mobile app on the mental health status of healthcare workers and providers of social services during the COVID-19 pandemic. The YOI intervention consisted of twice daily 30-min sessions, with a new session provided each week. Improvements in anxiety, depression, and insomnia were tracked with standard clinical questionnaires. This trial in healthcare workers was part of a larger clinical study of the YOI program in healthy adults (19, 20).

The working hypothesis for this study was that the combination of breathwork, yogic practices and meditation will have a significant and positive effect of the mental health of healthcare workers who participated in this intervention. The specific population of healthcare workers is the focus of this paper due to the importance of finding ways to ensure providers themselves do not become overwhelmed, burned out or exhausted during the intense workload of a global pandemic.

## Materials and methods

### Participants recruitment and grouping

The data presented here are a subset from a larger study of YOI intervention and its effect on mental health (19). The larger study used convenience sampling to recruit a large population of adults from Asia, North America, and Europe. Study participants were recruited through social media links distributed on Facebook, Twitter, and other social media platforms. Participants were sent a link to download the mobile application onto their personal phones and during the study all materials and instructions for the YOI interventions were delivered through the mobile app. A total of 1,500 possible eligible subjects were initially identified, which included adults aged 18–85 from a variety of countries. The initial pool of subjects was randomly assigned either to the “test” or “control” group. For specific demographics of the study population the sample of healthcare workers were recruited from see Verma et al. (19).

The sample of healthcare providers were thus drawn from the overall study population described in the prior study, by selecting participants based on their reported occupation. The healthcare worker sample from this study included a total of 445 participants, of which 151 were from the “test” group and 294 were from the “control” group. A “health care provider” was defined as any of the following professionals who are authorized to practice by the state, including: Doctor of Medicine (MD), Nurse (RN, e.g.), podiatrist, dentist, chiropractor, clinical psychologist, optometrist, nurse practitioner or a clinical social worker who is authorized to practice by the state. Other inclusion criteria included: adults aged 18 years of older, able to read and agree to informed consent page.

Since the study used a convenience sample design, we initially aimed for 200 subjects per group, as an initial power analysis showed that at *n* = 200 per group, we had a power of > 75% to detect a change of at least 2 points on any of the scored questionnaires used in the study, which corresponds to a medium effect size (Cohen’s *d* of 0.5–0.8). With the final group sizes of 151 and 294, our achieved power was 82% to detect a change of 3 points or 30% minimum change. As will be shown in the results, the average detected changes in the study exceeded 30%, and so the results are adequately powered, given the non-randomly drawn convenience sample.

Prior to the intervention, all participants were asked to complete the following questionnaires: (1) the Patient Health Questionnaire-8 (PHQ-8), (2) the Generalized Anxiety Disorder 7-item (GAD-7) scale, and (3) the Insomnia Severity Index (ISI). Baseline assessment also included the following demographic variables: age, race/ethnicity, marital and employment status, education, and occupation. In addition, participants were asked to report if they had any chronic medical conditions.

Participants who were in the “test” (YOI) group had access to the YOI app and completed twelve weeks of the intervention. Those in the control group were provided one weekly educational article on mental well-being. The control group was not made aware of the YOI app intervention. Both groups were asked to complete the mental health questionnaires.

### Yoga of Immortals intervention

The YOI intervention consisted of daily sessions delivered over twelve weeks. The sessions changed weekly. Prior to the beginning of each week, participants were asked to read and watch the associated instruction in preparation for the weekly sessions. Each individual session lasted for approximately 30 min. The protocol included two sessions a day in the morning and evening. The morning sessions included a combination of whole-body movements, postures, and yogic breathwork (cyclical controlled breathing practices including abdominal-pelvic breathing) synchronized with meditation and chants. The evening sessions included slow, deep yogic breathwork and meditations. With each week, the sessions became more advanced, building upon the work of prior weeks.

The mental health questionnaires, described below, were provided to participants via email. All participants were asked to complete the three screening questionnaires at baseline, after eight weeks, and again after twelve weeks. Participants were also asked to complete a study survey at the end of their participation in the study. To ensure data quality, human verification and attention checks were implemented throughout the survey; the data was further inspected visually for response irregularities potentially indicative of automated “bots.”

### General anxiety disorder scale

The GAD-7 is a self-administered 7-item scale used to screen for generalized anxiety disorder. The GAD-7 may be particularly useful in assessing symptom severity and monitoring change across time. The diagnostic validity of the GAD-7 is high (Cronbach’s alpha of 0.79–0.91) (28). Cut points of 5, 10, and 15 may be interpreted as representing mild, moderate, and severe levels of anxiety. This means a total score range of 0–4 indicates no anxiety; 5–9: mild anxiety; 10–14: moderate anxiety; and 15–21: severe anxiety. A score of 10 or greater on the GAD-7 represents a reasonable cut point for identifying cases of GAD (28).

### Insomnia severity index

Insomnia severity was assessed using the Insomnia Severity Index (ISI), an instrument posing seven questions to assess current (i.e., preceding 2 weeks) sleep characteristics, with a good diagnostic validity (0.85–0.9) Cronbach’s alpha (29, 30). The first three items pose questions related to sleep onset, sleep maintenance, and early morning awakening. Subsequent items assess the degree of satisfaction or dissatisfaction with the current sleep pattern, how the current sleep pattern interferes with daily functioning, how noticeable the impairment attributed to the sleep problem is, and how worrisome is the current sleep problem. Items were rated on a five-point Likert scale (“0” representing none or not at all and “4” representing very much). Total scores ranged from 0 to 28, with higher combined scores indicating worse insomnia severity (29). Participants were placed in total score groups as follows: 0–7 = no clinically significant insomnia; 8–14 = subthreshold insomnia; 15–21 = clinically significant insomnia (moderate); 22–28 = clinically significant insomnia (severe).

### Patient health questionnaire

Symptoms of depression were assessed using the 8-item version of the Patient Health Questionnaire (PHQ-8) (30). The PHQ-8 version used was the standardized and modified response set of Kroenke et al. (31), and is reported to have a Cronbach’s alpha of 0.86–0.91 (31–33). Current depression symptoms were defined as a PHQ-8 score of ≥ 10 (32) which, regardless of diagnostic status, typically correlates with clinically significant depression (31, 33).

### Statistical analysis

All statistical analysis was done using the Statistical Package for Social Science (SPSS) and Prism (Graphpad). Data from the three questionnaires fit the assumption of normality (*p* > 0.1, Shapiro-Wilk test) and were treated as normally distributed interval (34). The ISI, PHQ8 and GAD-7 data are expressed in data tables and text as mean ± SEM (standard error of mean).

Paired t tests were applied for within group comparisons. Chi-squared test was applied for comparison of percentage scores between pre- and post-intervention scales. Mixed effects analysis (Chi square test and *p* value summary) with Sidak’s multiple comparisons on selected pairs of groups was applied for comparison of results between week 0 control, 6 month, week 0 YOI and 6 month YOI. For all tests, a *p* value < 0.05 (two-sided) was considered statistically significant.

To evaluate for changes in mean scores as well as subitem scores across all grouping factors, we used two-way ANOVA to test for factors that significantly contributed to between group differences (participant vs. control) and within group differences (pre vs post intervention). The normalized mean scores or means for each subitem score served as the main dependent variable. *Post hoc* tests using Tukey’s honest standardized differences method to correct for multiple comparisons were used to compare across each level of both factors following the initial ANOVA. As with the previous analyses, the threshold was set at *p* < 0.05.

### Institutional review board approval

The study was approved by the Institutional Review Board, University of Cincinnati. Informed consent was obtained from all participants included in this study.

## Results

### Demographics analysis

**FIGURE 1:**
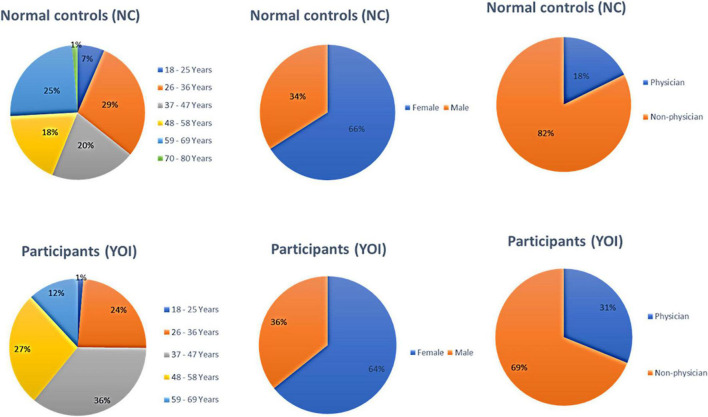
Demographic characteristics of the control and participants groups.

A total of 445 participants completed all aspects of the study (151 participants in the YOI group and 294 controls). Demographic data are provided in Table 1 and Figure 1.

**TABLE 1:** Demographic details of the study population in numbers as well as percentage.

Parameters	Categories	Controls		Test group (YOI)	
		Numbers (Total 294)	Percentage	Numbers (Total 151)	Percentage
Age	18–25 years	19	6.46	2	1.32
	26–36 years	86	29.25	36	23.84
	37–47 years	60	20.41	54	35.76
	48–58 years	53	18.03	41	27.15
	59–69 years	72	24.49	18	11.92
	70–80 years	4	1.36	0	0
	>80 years	0	0	0	0
Gender	Female	194	65.99	97	64.24
	Male	100	34.01	54	35.76
Race and ethnicity	American Indian or Alaska Native	3	1.02	1	0.66
	Asian	234	79.59	137	90.73
	White	43	14.63	10	6.62
	Other	14	4.76	3	1.99
Category by profession	Physician	52	17.69	47	31.13
	Non-physician	242	82.31	104	68.87
Self-reported associated chronic medical conditions	Arthritis	2	0.68	9	5.96
	Chronic pain	14	4.76	14	9.27
	Fibromyalgia	2	0.68	3	1.99
	Irritable bowel disease (IBD)	1	0.34	1	0.66
	Chronic Pain, Arthritis	1	0.34	1	0.66
	Chronic pain, fibromyalgia	1	0.34	0	0
	Chronic Pain, IBD	0	0	3	1.99
	Fibromyalgia, Arthritis	0	0	1	0.66
	Chronic Pain, Fibromyalgia, Arthritis	0	0	1	0.66
	None of the above	273	92.86	118	78.15

### Generalized anxiety disorder-7

**FIGURE 2:**
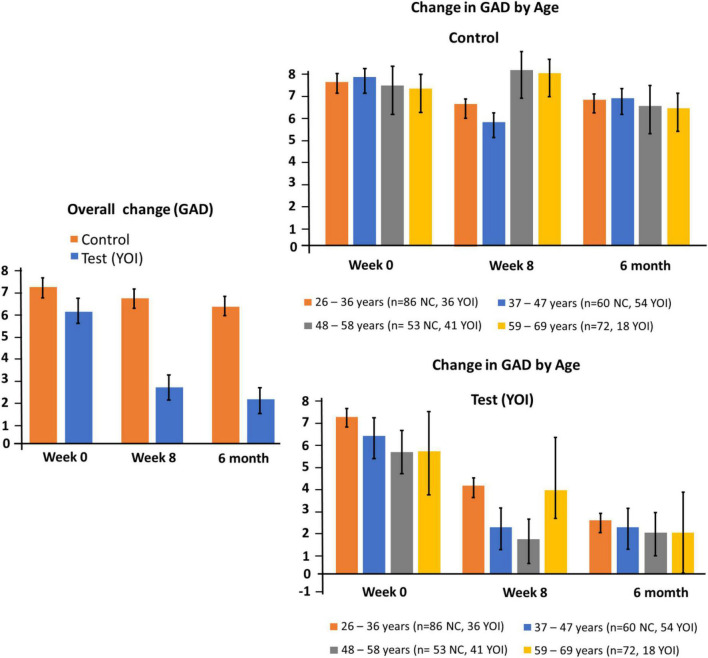
Changes in GAD-7 at week 0, 8 and 20. Left panel: overall change for controls and participants. Right panel: subgroup changes by age.

Table 2 and Figure 2 show the overall mean total GAD-7 scores as well as the mean total scores by demographic sub-groups. The overall mean total GAD-7 score at Week 0 in the control group (*n* = 294) was 5.03 ± 0.29. At 6 months, the mean total GAD-7 score was 4.51 ± 0.24. The difference between the two means was not statistically significant (Table 2). The mean total GAD-7 score at Week 0 in the YOI group (*n* = 151) was 5.01 ± 0.42. At week 8, the mean total ISI score was 2.11 ± 0.25. The improvement in the GAD-7 scores in week twelve was statistically significant (*p* < 0.001) as compared to week 0 in the YOI group. Statistical significance in the difference between the mean total GAD scores was also seen between week twelve control and week twelve YOI (*p* < 0.0001), with week 8 YOI group showing lower mean GAD-7 scores than week twelve control group. Comparable reductions were observed regardless of whether healthcare workers reported any chronic medical conditions.

**TABLE 2:** Overall mean total GAD-7 score and mean total GAD-7 scores for demographic parameters in control and YOI group, at week 0 and after completing 20 weeks of the YOI program.

Parameters	Categories	Controls		Test (YOI)		Mixed effect analysis		
		Week 0 (Mean ± SEM)	6 months (Mean ± SEM)	Week 0 (Mean ± SEM)	6 months (Mean ± SEM)	Between columns- Fixed value *P*	Chi -square	*P*-value summary
Overall		5.03 ± 0.29	4.51 ± 0.24	5.01 ± 0.42	2.11 ± 0.25**** $$$$	<0.0001	25.9	<0.0001
Age	18–25 years (*n* = 19 control, 2 YOI)	Statistics not done due to less numbers in YOI						
	26–36 years (*n* = 86 control, 36 YOI)	6.09 ± 0.6	4.72 ± 0.47	7.14 ± 0.96	2.06 ± 0.64**	<0.01	16.91	<0.0001
	37–47 years (*n* = 60 control, 54 YOI)	4.53 ± 0.54	3.88 ± 0.46	5.69 ± 0.75	1.91 ± 0.32**** $$	<0.0001	4.48	<0.05
	48–58 years (*n* = 53 control, 41 YOI)	3.38 ± 0.52	4.47 ± 0.58	3.71 ± 0.57	1.46 ± 0.29**** $$$	<0.01	0.05	>0.05
	59–69 years (*n* = 72 control, 18 YOI)	5.88 ± 0.61	5.72 ± 0.49	3.72 ± 1.37	2.56 ± 1.12	<0.01	20.98	<0.0001
	70–80 years (*n* = 4 control, 0 YOI)	Statistics not done due to less numbers						
Gender	Female (*n* = 194 control, 97 YOI)	4.93 ± 0.34	4.83 ± 0.31	4.94 ± 0.52	1.96 ± 0.28**** $$$$	<0.0001	23.9	<0.0001
	Male (*n* = 100 control, 54 YOI)	5.23 ± 0.52	4.19 ± 0.37	5.28 ± 0.73	2.39 ± 0.49*** $	<0.001	10.63	<0.01
Category by profession	Physician (*n* = 52 control, 47 YOI)	4.89 ± 0.63	4.25 ± 0.56	3.72 ± 0.68	1.71 ± 0.31** $$$	<0.01	3.76	>0.05
	Non-physician (*n* = 242, 104)	5.07 ± 0.32	4.69 ± 0.27	5.64 ± 0.61	2.32 ± 0.34**** $$$$	<0.0001	38.95	<0.0001
Self-reported associated chronic medical conditions	Chronic medical conditions (*n* = 21 control, 33 YOI)	5.33 ± 1.1	4.91 ± 0.86	7.55 ± 1.04	3.24 ± 0.78****	<0.01	11.8	<0.001
	No chronic medical conditions (*n* = 273 control, 118 YOI)	5.01 ± 0.3	4.59 ± 0.25	4.36 ± 0.43	1.78 ± 0.23**** $$$$	<0.0001	53.73	<0.0001
	Others (*n* = 37 control, 12 YOI)	5.24 ± 0.72	5.54 ± 0.79	7.33 ± 1.5	3.17 ± 1.3**	>0.05	2.82	>0.05
	None of the above (*n* = 35 control, 6 YOI)	Statistics not done due to less numbers in control						

Adjusted *p* (vs week 0 YOI * < 0.05, ** < 0.01, *** < 0.001, **** < 0.0001; vs week 8 control ^$^ < 0.05, ^$$^ < 0.01, ^$$$^ < 0.001, ^$$$$^ < 0.0001) in mixed effects analysis with Sidak’s multiple comparisons test.

### Insomnia severity index

Table 3 shows the overall mean total ISI scores as well as the mean total scores by demographic subgroups. The mean total ISI score at week 0 in the control group (*n* = 294) was 7.26 ± 0.45. At week twelve, the mean total ISI score was 6.84 ± 0.40. The mean total ISI score at Week 0 in the YOI group (*n* = 151) was 6.23 ± 0.58. At week twelve, the mean total ISI score was 2.5 ± 0.32.

**TABLE 3:** Overall mean total ISI score and mean total ISI scores for demographic parameters in control and YOI group.

Parameters	Categories	Controls		Test (YOI)		Mixed effect analysis		
		Week 0 (Mean ± SEM)	6 months (Mean ± SEM)	Week 0 (Mean ± SEM)	6 months (Mean ± SEM)	Between columns- Fixed value *P*	Chi -square	*P*-value summary
Overall		7.3 ± 0.45	6.8 ± 0.4	6.2 ± 0.58	2.5 ± 0.32**** $$$$	<0.0001	77.22	<0.0001
Age	18–25 years (*n* = 19 control, 2 YOI)	Statistics not done due to less numbers in YOI						
	26–36 years (*n* = 86 control, 36 YOI)	7.71 ± 0.88	6.56 ± 0.7	7.36 ± 1.23	3.11 ± 0.85** $	<0.05	51.16	<0.0001
	37–47 years (*n* = 60 control, 54 YOI)	7.83 ± 0.98	5.78 ± 0.76	6.43 ± 0.95	1.2 ± 0.43**** $$$	<0.0001	18.87	<0.0001
	48–58 years (*n* = 53 control, 41 YOI)	7.4 ± 1.1	8.09 ± 1.15	5.75 ± 1.01	1.49 ± 0.4*** $$$$	<0.001	14.04	<0.001
	59–69 years (*n* = 72 control, 18 YOI)	7.25 ± 0.88	7.97 ± 0.82	5.72 ± 1.96	2.94 ± 1.31	>0.05	16.54	<0.0001
	70–80 years (*n* = 4 control, 0 YOI)	Statistics not done due to less numbers						
Gender	Female (*n* = 194 control, 97 YOI)	6.72 ± 0.5	6.96 ± 0.47	6.32 ± 0.76	2.48 ± 0.39*** $$$$	<0.0001	32.92	<0.0001
	Male (*n* = 100 control, 54 YOI)	8.31 ± 0.89	6.54 ± 0.73#	6.06 ± 0.89	1.7 ± 0.58*** $$$	<0.01	42.39	<0.0001
Category by profession	Physician (*n* = 52 control, 47 YOI)	6.4 ± 0.93	5.87 ± 0.73	4.28 ± 0.95	1.06 ± 0.40* $$$$	<0.001	4.97	<0.05
	Non-physician (*n* = 242, 104)	7.44 ± 0.50	7.05 ± 0.46	7.04 ± 0.69	2.04 ± 0.43**** $$$$	<0.0001	78.65	<0.0001
Self-reported associated chronic medical conditions	Chronic medical conditions (*n* = 21 control, 33 YOI)	7.62 ± 1.79	7.91 ± 1.52	10.21 ± 1.54	2.46 ± 0.79**** $	<0.001	21.83	<0.0001
	No chronic medical conditions (*n* = 273 control, 118 YOI)	7.23 ± 0.46	6.76 ± 0.41	5.11 ± 0.57##	1.47 ± 0.35**** $$$$	<0.0001	112.0	<0.0001

Adjusted *p* (vs week 0 control ^#^ < 0.05; vs week 0 YOI * < 0.05, ** < 0.01, *** < 0.001, **** < 0.0001; vs week 8 control ^$^ < 0.05, ^$$^ < 0.01, ^$$$^ < 0.001, ^$$$$^ < 0.0001) in mixed effects analysis with Sidak’s multiple comparisons test.

The improvement in the ISI scores week twelve was statistically significant (*p* < 0.0001 in Sidak’s multiple comparisons test) as compared to week 0 in the YOI group. Statistical significance in the difference between the mean total ISI scores was also seen between week twelve control and week twelve YOI (*p* < 0.0001), with week twelve YOI group showing better ISI scores than week twelve control group.

**FIGURE 3:**
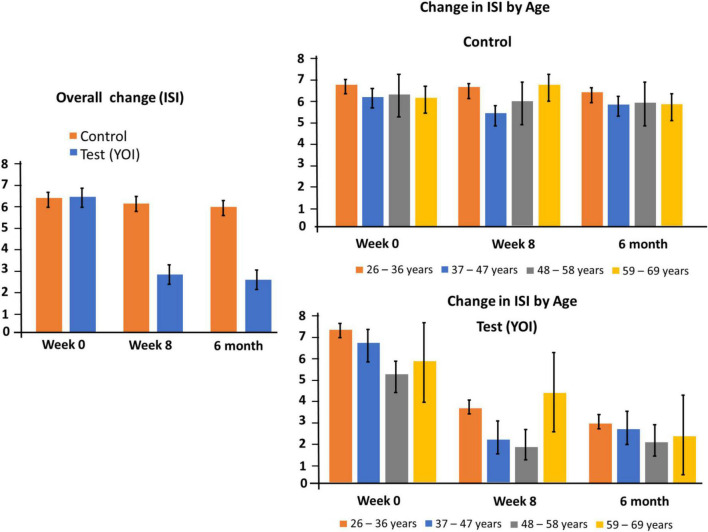
Changes in ISI at week 0, 8 and 20. Left panel: overall change for controls and participants. Right panel: subgroup changes by age.

These results indicate that the ISI score improvement was better in the YOI group than in the control group. Comparable results were seen in the demographic sub-groups (Table 3 and Figure 3) – the YOI interventions significantly improved ISI scores in all age groups, regardless of gender, and in physicians as well as non-physicians. YOI benefitted healthcare workers even if they self-reported chronic medical conditions.

All the above results indicate that the YOI intervention of twelve weeks improved the ISI scores and sleep patterns in the study population in general, as well as within the different demographic sub-groups.

### Depression symptom severity assessment

**FIGURE 4:**
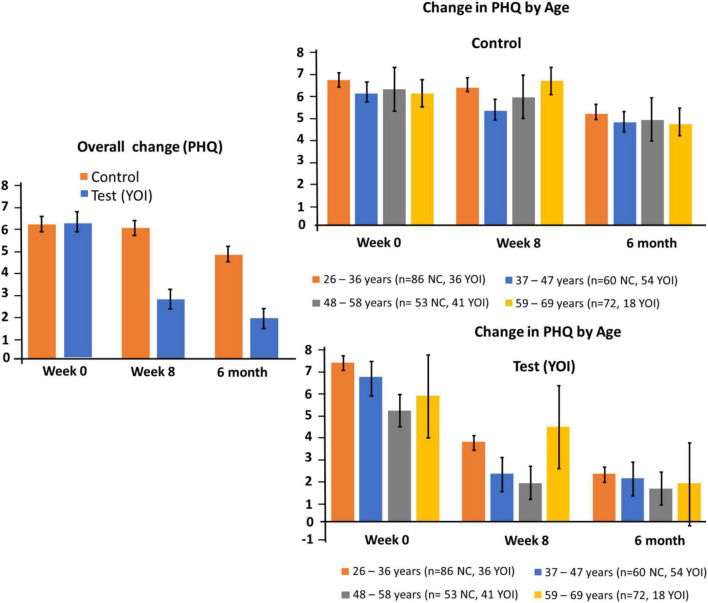
Changes in PHQ-8 at week 0 and Week 8. Left panel: overall change for controls and participants. Right panel: subgroup changes by age.

Table 4 and Figure 4 show the overall mean total PHQ-8 scores as well as the mean total scores by demographic sub-groups. In the PHQ-8 assessment scale, the mean total score of control for week 0, (*n* = 294) was 6.3 ± 0.34. At week twelve, the mean total was 5.9 ± 0.31. The mean total score of YOI for week 0, (*n* = 151) was 6.4 ± 0.47. At week twelve, the mean total was 1.6 ± 0.34. The improvement in the PHQ-8 scores week twelve was statistically significant (*p* < 0.0001 in Sidak’s multiple comparisons test) as compared to week 0 in the YOI group. Statistical significance in the difference between the mean total PHQ-8 scores was also seen between week 8 control and week 8 YOI (*p* < 0.0001), with week 8 YOI group showing better PHQ-8 scores than week 8 control group.

**TABLE 4:** Overall mean total PHQ-8 score and mean total PHQ-8 scores for demographic parameters in control and YOI group.

Parameters	Categories	Controls		Test (YOI)		Mixed effect analysis		
		Week 0 (Mean ± SEM)	6 months (Mean ± SEM)	Week 0 (Mean ± SEM)	6 months (Mean ± SEM)	Between columns- Fixed value *P*	Chi -square	*P*-value summary
Overall		6.3 ± 0.34	5.9 ± 0.31	6.4 ± 0.47	1.6 ± 0.34**** ^$$$$^	<0.0001	55.19	<0.0001
Age	18–25 years (*n* = 19 control, 2 YOI)	Statistics not done due to less numbers in YOI						
	26–36 years (*n* = 86 control, 36 YOI)	6.8 ± 0.66	4.8 ± 0.63	7.53 ± 0.91	1.55 ± 0.76**** ^$^	<0.05	26.11	<0.0001
	37–47 years (*n* = 60 control, 54 YOI)	6.23 ± 0.68	5.8 ± 0.63	6.78 ± 0.8	1.14 ± 0.43**** ^$$$^	<0.0001	9.61	<0.01
	48–58 years (*n* = 53 control, 41 YOI)	6.36 ± 1.02	6.23 ± 0.67	5.27 ± 0.76	1.21 ± 0.33**** ^$$$$^	<0.01	9.74	<0.01
	59–69 years (*n* = 72 control, 18 YOI)	6.17 ± 0.63	6.01 ± 0.63	5.94 ± 1.95	3.5 ± 1.8	>0.05	15.7	<0.0001
	70–80 years (*n* = 4 control, 0 YOI)	Statistics not done due to less numbers						
Gender	Female (*n* = 194 control, 97 YOI)	5.89 ± 0.38	6.21 ± 0.39	6.56 ± 0.61	1.83 ± 0.42**** ^$$$$^	<0.0001	32.55	<0.0001
	Male (*n* = 100 control, 54 YOI)	7.16 ± 0.68	6.65 ± 0.5^#^	6.11 ± 0.76	1.57 ± 0.57**** ^$$^	<0.001	24.34	<0.0001
Category by profession	Physician (*n* = 52 control, 47 YOI)	4.64 ± 0.68	4.31 ± 0.65	3.92 ± 0.63	1.6 ± 0.44**** ^$$$$^	<0.001	13.28	<0.001
	Non-physician (*n* = 242, 104)	6.68 ± 0.39	6.51 ± 0.35	7.48 ± 0.59	2.1 ± 0.44**** ^$$$$^	<0.0001	53.88	<0.0001
Self-reported associated chronic medical conditions	Chronic medical conditions (*n* = 21 control, 33 YOI)	6.19 ± 1.11	7.35 ± 1.46	9.12 ± 1.26	3.18 ± 1.11****	<0.01	21.55	<0.0001
	No chronic medical conditions (*n* = 273 control, 118 YOI)	6.33 ± 0.36	4.94 ± 0.31	5.64 ± 0.48	1.56 ± 0.29**** ^$$$$^	<0.0001	86.15	<0.0001

Adjusted *p* (vs week 0 control ^#^ < 0.05; vs week 0 YOI * < 0.05, ** < 0.01, *** < 0.001, **** < 0.0001; vs week 8 control ^$^ < 0.05, ^$$^ < 0.01, ^$$$^ < 0.001, ^$$$$^ < 0.0001) in mixed effects analysis with Sidak’s multiple comparisons test.

These results show that the PHQ-8 score improvement was better in the YOI group than in the control group, at week 8. Equivalent results were seen in the demographic sub-groups (Table 4) – the YOI interventions significantly improved ISI scores at week twelve in all age groups at week twelve, regardless of gender, and in physicians as well as non-physicians

Table 4 shows healthcare workers with self-reported chronic medical conditions seemed to benefit from the YOI intervention in terms of statistically significantly improved PHQ-8 scores at week twelve, as shown by the *p* values of week 0 YOI versus week twelve YOI as well as week twelve control versus week twelve YOI.

### Generalized anxiety disorder 7, insomnia severity index and patient health questionnaire-8 score ranges

Table 5 shows the number of individuals in each of the score ranges of the GAD, ISI and PHQ8 measures at week 0 and week 8 in the control (total *n* = 294) and YOI (*n* = 151) groups. At week 0 there was a larger fraction of individuals in the mild, moderate, or severe categories of GAD-7 and ISI than in the clinically normal groups of GAD-7 or ISI. At week 8, there were more individuals in the normal groups than in the clinically affected groups of GAD-7, ISI, or PHQ-8. The control group demonstrated mixed results in terms of increase of number of individuals in the clinically affected groups at week 8 (Table 5).

**TABLE 5:** Number of individuals in each of the score ranges of GAD, ISI and PHQ8 at week 0 and week 8 in controls (total *n* = 294) and test (*n* = 151) groups.

Parameters	Score range	Control (*n* = 294)				YOI (*n* = 151)			
		Number of individuals		™	™ as% of week 0	Number of individuals		™	™ as% of week 0
		Week 0	6 months			Week 0	6 months		
GAD-7	None (0–5)	185	186	1	0.54% worse	97	144	37	38.14% better
	Mild (6–10)	66	78	12	18.19% worse	30	10	18	60% better
	Moderate (11–15)	28	27	1	3.57% better	15	4	11	73.33% better
	Severe (16–21)	15	5	10	66.67% better	9	1	8	88.89% better
ISI	No clinically significant Insomnia (0–7)	188	196	8	4.26% better	105	139	34	32.38% better
	Mild (8–14)	50	52	2	4.0% worse	21	6	15	71.43% better
	Moderate (15–21)	33	31	2	6.06% better	19	6	13	68.42% better
	Severe (22–28)	23	15	8	34.78% better	6	0	6	100% better
PHQ-8 for current depression symptoms	No major depression (< 10)	221	221	0	No change	111	142	31	27.92% better
	Major depression (≥ 10)	73	73	0	No change	40	9	31	77.5% better

™ = difference in numbers between week 0 and week 8 within each group.

Table 5 displays PHQ-8 depressive symptoms based on the cut-off total score of 10 in both control and YOI groups. There were no changes in current depression status in the < 10 or ≥ 10 category in control. However, in the YOI group, 77.5% of the individuals who had their PHQ-8 scores ≥ 10 at week 0, had the scores improve to < 10 at week 8, indicating marked improvement in depression symptoms after 8 weeks of YOI intervention.

## Discussion

Depression, anxiety, and insomnia symptoms amongst healthcare workers increase during public health crises. The COVID-19 pandemic has been the largest global crisis in a century, leading to huge, overwhelming caseloads of infected patients, periods of personal protective equipment scarcity, workforce shortages, persistent threats of viral exposure, and fears of infecting family and friends outside of healthcare settings, just to name a few of stresses it has caused. The emotional impact on healthcare workers has been well documented, and appropriate interventions are clearly needed.

In this study, we found large (often > 50%) and statistically significant reductions in scores on clinically validated mental health scales among participants who completed the YOI program, but not among control participants. This effect was observed for measures of anxiety, depression, and insomnia. Thus, the trial provides evidence that the YOI intervention can provide a meaningful benefit for those in the healthcare field that suffer from depression, anxiety, and sleep issues. Furthermore, the results were consistently positive when subdivided into demographic and age-based subgroups, suggestive of widespread applicability.

Improvements were seen amongst those in the severe range of symptoms, further suggesting that YOI may be effective regardless of symptom intensity. The number of participants who reported moderate or severe symptoms decreased significantly after 8 weeks, and further decreased after 20 weeks. Furthermore, at both 8 and 20 weeks, the number of participants with severe symptoms were significantly less than the number of participants with mild or no symptoms. Lastly, there was a notable improvement of PHQ-8 and GAD scores in healthcare workers with self-reported chronic medical conditions (for example inflammatory bowel disease) which suggests that the YOI intervention could have benefits for those with both physical and psychological symptoms.

The YOI program is a unique combination of very deliberate and specific breath work, meditation, physical movements, and postures that have their origins in ancient teachings, examining its effectiveness in mitigating depression, anxiety and insomnia in the background of a pandemic. The founders of this program maintain that the specific composition of techniques, as well as their precise arrangements within the program, will have a much greater benefit than if the individual components were to be practiced independently. Thus, finding comparison studies of similar programs has been challenging.

This study of YOI in a population of healthcare workers contributes significantly to the literature, which has thus far has focused on specific yoga practices done in isolation, or use a mix of yoga and mindfulness, and in the general adult population. Several practices have shown positive benefits for mental health, although results are mixed. A recent systematic review yielded mixed results for use of yoga as an adjunct treatment (35). In contrast, mindfulness-based interventions have shown some benefit in insomnia (36) but low yield results for depression (36). Nevertheless, a body of evidence has developed over the past two decades that shows some evidence for positive effects beyond placebo as an adjunct treatment for clinical depression and sleep disorders (37, 38). One of the other core features of YOI protocol is breath work, including abdominal breathing, and several studies have shown that abdominal breathing could decrease depressive symptoms and reduce cortisol levels (39). Abdominal breathing may serve as a bridge linking the autonomic nervous system and the central nervous system to mobilize vagal activation of GABA (gamma-aminobutyric acid) pathways from the prefrontal cortex and insula, and to inhibit amygdala overactivity (40) leading to decreased depressive symptoms.

Therefore, several of the components or practices like that used in the YOI intervention have been studied and shown some benefit, with breathwork possibly acting through a physiological mechanism involving the autonomic nervous system, while yoga and mindfulness have had success in reducing levels of anxiety and depression. Therefore, it was of interest to examine if the practices of yoga, breathwork and mindfulness as it is structured in the YOI intervention, would be of greater benefit, in a specific population which requires more flexible, low-cost interventions for mental health. We hypothesis that in the current study, the cause of the observed improvements in depression, anxiety and insomnia may be linked to the same physiological benefits of breathwork and associated mediative practices.

### Limitations and future directions

However, future studies will be needed to further elucidate the specific extent of the potential benefits provided by YOI. The initial set of studies (19, 20) used self-reported questionnaires, which have an inherent bias, as the subjects may exaggerate or minimize specific symptoms. However, such self-reported questionnaires are routinely used in psychology studies and are well-validated instruments. The study relied on participant’s willingness to adhere to the intervention and fill out all questions. The use of a control group reduced this bias. Additionally, the surveys did not ask for weight or BMI information, or details on sleep hygiene. High BMI can lead to sleep apnea and poor sleep hygiene contribute to disrupted sleep, and these factors, if present in our study population, could have affected the results. Participant connection to the app may have an indirect beneficial effect toward alleviating the loneliness and social isolation contributing to symptoms of anxiety or insomnia in some.

Further studies using the YOI intervention are ongoing and so future studies will strive to collect more detailed and nuanced information. A potential future direction will be to compare YOI with other yoga mindfulness practices. YOI could be compared to other interventions that involve physical activity, to see if the specific type of physical activity indicated by YOI contributes to the effect seen on depression and anxiety scores. This will enable more direct comparisons between the different components of YOI and other interventions.

## Conclusion

Healthcare workers face unique demands during the COVID-19 pandemic, and face greater risk for depression, anxiety, and disrupted sleep. The YOI intervention provides a unique combination of breath work, yoga and meditation that together produce a measurable benefit in this group most challenged by the pandemic. It can be safely accessed from anywhere, through a digital app, and can be utilized at the convenience of the health care worker, in a socially distanced manner.

## Data availability statement

The original contributions presented in this study are included in the article/supplementary material, further inquiries can be directed to the corresponding author.

## Ethics statement

The studies involving human participants were reviewed and approved by Institutional Review Board, University of Cincinnati, Cincinnati, Ohio. IRB approval no: 2020-0494. The patients/participants provided their written informed consent to participate in this study.

## Author contributions

KC: data curation, original draft preparation, and software. SV: conceptualization, methodology, formal analysis, and writing. IS: review and editing and supervision. AD: conceptualization, writing, and software. SB: methodology and writing – review. HT: conceptualization and data curation. BG: conceptualization, methodology, and writing – review and editing. All authors have read and agreed to the published version of the manuscript.
